# Nesfatin‐1 inhibits cerebral aneurysms by activating Nrf2 and inhibiting NF‐κB signaling

**DOI:** 10.1111/cns.14864

**Published:** 2024-08-04

**Authors:** Huimin Yu, Qingyuan Liu, Minghong Xie, Junquan Fan, Jiajia Luo, Junping Huang, Lei Chen

**Affiliations:** ^1^ Department of Neurology, The First Dongguan Affiliated Hospital Guangdong Medical University Dongguan China; ^2^ Department of Neurosurgery, Beijing Tiantan Hospital, China National Clinical Research Center for Neurological Diseases Capital Medical University Beijing China; ^3^ Department of Neurosurgery, The First Dongguan Affiliated Hospital Guangdong Medical University Dongguan China; ^4^ Department of Neurosurgery Minzu Hospital of Guangxi Zhuang Autonomous Region Nanning China

**Keywords:** cerebral aneurysm, inflammation, macrophage, Nesfatin‐1, NF‐κB

## Abstract

**Aims:**

Cerebral aneurysm (CA) has been considered one of the most common cerebrovascular diseases, affecting millions of people worldwide. A therapeutic agent is currently missing for the treatment of CA. Nesfatin‐1 (Nes‐1) is an 82‐amino acid adipokine which possesses a wide range of biological functions. However, the physiological function of Nes‐1 in CA is still unknown. Here, we aimed to assess the preventive effects of Nes‐1 in the pathological development of CA and elucidate the mechanisms behind this.

**Methods:**

We used an elastase‐induced CA model, accompanied by a high‐salt diet to induce hypertension. Additionally, diverse experimental techniques, including Verhoeff‐Van Gieson staining, real time PCR, enzyme‐linked immuno sorbent assay (ELISA), and immunofluorescence staining, were employed to assess CA formation, gene and protein expression, as well as the macrophage infiltration.

**Results:**

Our results indicate that administration of Nes‐1 significantly decreased the aneurysm size. Additionally, Nes‐1 prevented inflammatory response by inhibiting the expression of interleukin‐6 (IL‐6), tumor necrosis factor‐α (TNF‐α), and monocyte chemoattractant protein 1 (MCP‐1) at both the mRNA and protein levels in the Circle of Willis (COW) region. Also, the increased levels of matrix metalloproteinase‐2 (MMP‐2) and matrix metalloproteinase‐9 (MMP‐9) in the COW region were reduced by Nes‐1. We found that Nes‐1 administration suppressed the invasion of macrophages. Mechanistically, Nes‐1 activated Nrf‐2 by promoting its nuclear translocation but prevented the activation of the IκBα/NF‐κB signaling pathway.

**Conclusion:**

These findings suggest that Nes‐1 might be used as a promising agent for the prevention of CA.

## Graphical abstract

This study shows that Nes‐1 activated Nrf‐2 by promoting its nuclear translocation but prevented the activation of the IκBα/NF‐κB signaling pathway to inhibit the expression of pro‐inflammatory mediators such as IL‐6, TNF‐α, MCP‐1, MMP‐2, and MMP‐9. Subsequently, Nes‐1 suppressed the invasion of macrophages and CA formation.

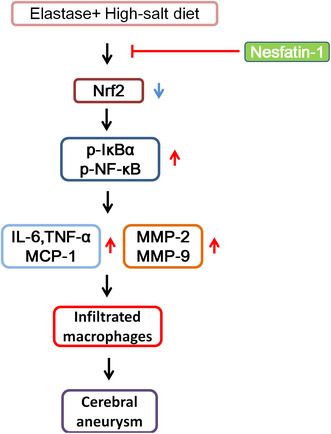

## 1 INTRODUCTION

Cerebral aneurysm (CA) refers to a change in the cerebral arterial wall caused by local thinning and bulging, mostly found in the Circle of Willis (COW) and its branches. It is a common cerebrovascular disease, and its incidence in the general population is as high as 3%–5%.^1^ Several complications, such as subarachnoid hemorrhage and cerebral hemorrhage, can be triggered by CA.^2^ Fortunately, the annual rupture rate of CA is only about 0.7%, and most CA patients will not experience any clinical symptoms throughout their lifetime.^3^ Although several indicators (such as the size, location, and shape) can help clinicians evaluate the rupture risk of CA, there is still a considerable proportion of low rupture risk small CA (diameter less than 5 mm) that ruptures.^4^ Thus, predicting the rupture risk of CA and seeking non‐invasive drug intervention methods are crucial for CA treatment. The formation of CA involves complex pathophysiological processes in addition to genetic factors, which are markedly related to endothelial inflammatory reactions caused by changes in blood flow dynamics.^5^ Changes in cerebral arterial hemodynamics trigger long‐term excessive inflammatory reactions in the vessel wall, leading to the formation, growth, and rupture of CA.^6^ Chronic inflammation involves the infiltration of macrophages/monocytes, neutrophils, and the release of inflammatory cytokines and related proteinases such as matrix metalloproteinases (MMPs), which induce vascular cell death and degradation of the extracellular matrix (ECM).^5^ Neutrophils are the first immune cells to migrate to the CA site to engulf and destroy pathogens or damaged cells through phagocytosis. With the growth of CA, the number of macrophages in the aneurysm wall increases, and widespread macrophage infiltration enhances ECM degradation, ultimately increasing the risk of CA rupture. T cells, mast cells, and humoral reactions are also involved in CA formation.^7^ The interaction between neutrophils, macrophages, and other immune cells is a critical pathological feature of CA. This interaction contributes to the chronic inflammation and remodeling of the blood vessel wall.^5^, ^6^

Santarosa et al.^8^ used high‐resolution vascular wall magnetic resonance imaging (VWMRI) to observe inflammatory cell infiltration in the vascular wall of CA, confirming the involvement of inflammation in CA. With the aggravation of inflammatory cell infiltration and endothelial dysfunction, the nuclear factor‐κB (NF‐κB) signal is activated, accompanied by elevated IL‐1β and TNF‐α levels. Subsequently, NF‐κB mediates the production of nitric oxide (NO) and pro‐inflammatory mediators, reactive oxygen species (ROS), cytokines, and cell adhesion molecules (CAM) to attack endothelial cells, ECM, and vascular smooth muscle cells (VSMCs), further causing endothelial damage, VSMC phenotypic switching, ECM remodeling, and Fas‐mediated cell apoptosis, and increasing the possibility of aneurysm rupture.^9^ Modulation of macrophage‐mediated chronic inflammation is an important target and the direction for CA treatment.

Nesfatin‐1 (Nes‐1) is an adipokine encoded by the *NEFA* gene and contains 82 amino acids.^10^ Nes‐1 is expressed in peripheral tissues including gastric mucosa, duodenum, pancreas, and central nervous system cortex.^11^, ^12^, ^13^ There is ample evidence to suggest that Nes‐1 is involved in the development of cardiovascular diseases.^14^, ^15^, ^16^ However, its impact on the progression of CA remains unclear. Recent studies show that Nes‐1 exerts a remarkably suppressive effect on endothelial inflammation,^17^ particularly regulating macrophage inflammatory reactions.^18^ Herein, we explored the potential role and underlying mechanism of Nes‐1 in CA. Our findings provided a potential preventive strategy for CA progression.

## 2 MATERIALS AND METHODS

### 2.1 CA modeling in rats and grouping

60 SD male rats were obtained from Shanghai Slack Laboratory Animal Co., LTD. For the CA modeling, rats were anesthetized with an intraperitoneal injection of 40 mg/kg pentobarbital sodium. Once the abdomen was opened, both renal arteries were separated and ligated with silk sutures. The left common carotid artery was also separated and ligated. The incision was sutured and disinfected, and rats were allowed to recover. During the 7 days after surgery, 50 g/L NaCl solution was used instead of drinking water. If rats showed symptoms of mild hemiplegia (slow movement, poor symmetry in movement, and weak extension of the unilateral forelimb) and oculomotor nerve palsy (deviation of the eye to one side and limited eye rotation), as well as increased intracranial pressure and body temperature, the model was considered successfully established. Animals were divided into four groups (*n* = 15/group): Control, CA, 10 μg/kg Nes‐1, and 20 μg/kg Nes‐1. Nes‐1 was administered intraperitoneally to rats 1 week before the induction of CA and continued until the end of the experiment, with animals in the control and CA groups being administered with the same volume of normal saline.

### 2.2 The measurement of the systolic blood pressure

The IITC rat blood pressure measurement system (USA) was utilized for the detection of rat tail systolic blood pressure.

### 2.3 Verhoeff‐Van Gieson staining

After fixation of Circle of Willis (COW) tissue in formaldehyde for 24 h, the tissue was washed with running water for 4 h, followed by immersed in different grades of alcohol, transparentized with absolute ethanol and xylene, immersed in paraffin, and cut into 5 μm sections. After heating for 90 min, slices were dewaxed. Verhoeff's working solution was added to the tissue for staining for 1 h. The sections were immersed in 2% FeCl_3_ solution for 1–2 min. After completion of the staining, sections were washed with 5% thiosemicarbazide solution for 5 min, followed by immersed in VG staining solution for 3–5 min. Sections were dehydrated with gradient alcohol (70%, 90%, 95%, and 100% ethanol in the water) and transparentized with xylene sealed with neutral gum and observed under the microscope (Mcalon, China). The size of the aneurysm was expressed as the average of the maximum longitudinal and transverse diameters.

### 2.4 Real‐time polymerase chain reaction (RT‐PCR)

The COW tissue was taken out and processed according to the RNA extraction kit manual, with total RNA extracted. The RNA was reversely transcribed into cDNA and stored for later use. A 20 μL PCR system was prepared, which included 1 μL TransScript RT/RI Enzyme Mix, 1 μL Anchored Oligo(dT)18 (0.5 μg/μL), 10 μL 2 × TS Reaction Mix, 1 μg total RNA, and 7 μL ddH_2_O. The reaction conditions were set at 94°C for 15 s, 60°C for 30 s, and 72°C for 30 s, and the signal was collected for 40 cycles, with error data excluded. The relative expression level was calculated using the 2^−△△CT^ method. The primers in this study are listed in Table 1.

**TABLE 1:** Primer sequences.

	Forward (5′–3′)	Reverse (5′–3′)
TNF‐α	5′‐AAATGGGCTCCCTCTCATCAGTTC‐3′	5′‐TCTGCTTGGTGGTTTGCTACGAC‐3′
IL‐6	5′‐AGACAGCCACTCACCTCTTCAG‐3′	5′‐TTCTGCCAGTGCCTCTTTGCTG‐3′
MCP‐1	5′‐CAGGTCTCTGTCACGCTTCT‐3′	5′‐AGTATTCATGGAAGGGAATAG‐3′
MMP‐2	5′‐GCTGATACTGACACTGGTACTG‐3′	5′‐CACTGTCCGCCAAATAAACC‐3′
MMP‐9	5′‐CTTGAAGTCTCAGAAGGTGGATC‐3′	5′‐CGCCAGAAGTATTTGTCATGG‐3′
GAPDH	5′‐CAACTCCCTCAAGATTGTCAGCAA‐3′	5′‐GGCATGGACTGTGGTCATGA‐3′

### 2.5 Enzyme‐linked immunosorbent assay (ELISA)

Rat TNF‐α (cat #ab46070), IL‐6 (cat #ab234570), and MCP‐1 (cat #ab219045) ELISA kits were purchased from Abcam (USA). Rat MMP‐2 (Cat #ERA42RB) and MMP‐9 (cat #EEL130) ELISA kits were purchased from Thermo Fisher Scientific (USA). The homogenate of the separated COW tissue was taken, and the content of TNF‐α, IL‐6, MCP‐1, MMP‐2, and MMP‐9 in the tissue was measured according to the double antibody sandwich method of the enzyme‐linked immunosorbent assay kit, which was also utilized for the detection of serum Nes‐1 level. The sample was added to the blank well, followed by diluted, sealed, and incubated for 30 min with a film. Working solution (100 μL) was added into each well and mixed well before incubation at 37°C for 60 min. The reaction was stopped, and the wavelength of the microplate reader (Tecan, Switzerland) was set to 630 nm to measure the absorbance value (value A). A standard curve was established with the control value A as the ordinate and the sample mass concentration as the abscissa. The sample concentration was calculated by substitution into the sample to be tested.

### 2.6 Immunofluorescence staining for detecting macrophage infiltration in aneurysmal tissue

The COW tissue was fixed in 4% paraformaldehyde for 24 h and then sliced into 30 μm sections with a cryostat. Sections were placed on a glass slide and dried overnight at room temperature. After antigen heat‐induced repair, 0. 01 M PBS was placed in a static state for 20 min for hydration, followed by being rinsed and blocked with 10% normal goat serum for 1 h. The mouse anti‐rat F4/80 primary antibody (1:200, CST, USA) was incubated. Then, the goat anti‐mouse secondary antibody (1:200, CST, USA) was introduced. After 4′,6‐diamidino‐2‐phenylindole (DAPI) staining and fixation, the infiltration of macrophages was observed under a fluorescent microscope (Zeiss, Germany).

### 2.7 Western blotting assay

The proteins in the cytoplasm and nucleus in the rat COW tissue were extracted after cutting the tissue into pieces using radio immunoprecipitation assay (RIPA) lysis buffer, respectively. The total protein concentration was determined using the bicinchoninic acid (BCA) method, and sodium dodecyl sulfate polyacrylamide gel electrophoresis (SDS‐PAGE) was performed. The proteins were transferred to a polyvinylidene fluoride (PVDF) membrane. After being sealed with 5% skim milk for 2 h, the corresponding primary antibody against Nrf‐2 (1:1000, #14596), NQO‐1 (1:1000, #62262), p‐IκBα (1:1500, #2859), IκBα (1:2000, #4812), p‐NF‐κB (1:500, # #3033), NF‐κB (1:2000, #8242), and Lamin B1 (1:2000, #13435) was purchased from Cell Signaling Technology (USA). The corresponding primary antibody against β‐actin (1:5000, #MAB8929) was purchased from R&D Systems (USA) as internal control. The reactions were incubated overnight at 4°C. The primary antibody was then washed off, and the secondary antibody was loaded to incubate for 60 min. The enhanced chemiluminescence (ECL) color was visualized using a gel imaging instrument to observe the protein bands and take pictures.

### 2.8 Statistical analysis

The data obtained were analyzed using GraphPad Prism 9.0. The normality of distribution of continuous variables was tested with a Kolmogorov‐Smirnov test, and the homogeneity of variance was assessed using Levene's test. Obtained data (mean ± SD) with a normal distribution were analyzed with a *t*‐test between two groups and analysis of variance (ANOVA) followed by Scheffe's test for multiple group comparisons. Data that do not exhibit a normal distribution from multiple group comparisons were conducted using a Kruskal‐Wallis test followed by a post hoc Dunn's test. The difference was statistically significant when *p* < 0. 05.

## 3 RESULTS

### 3.1 The levels of serum Nes‐1 were reduced in CA rats

**FIGURE 1:**
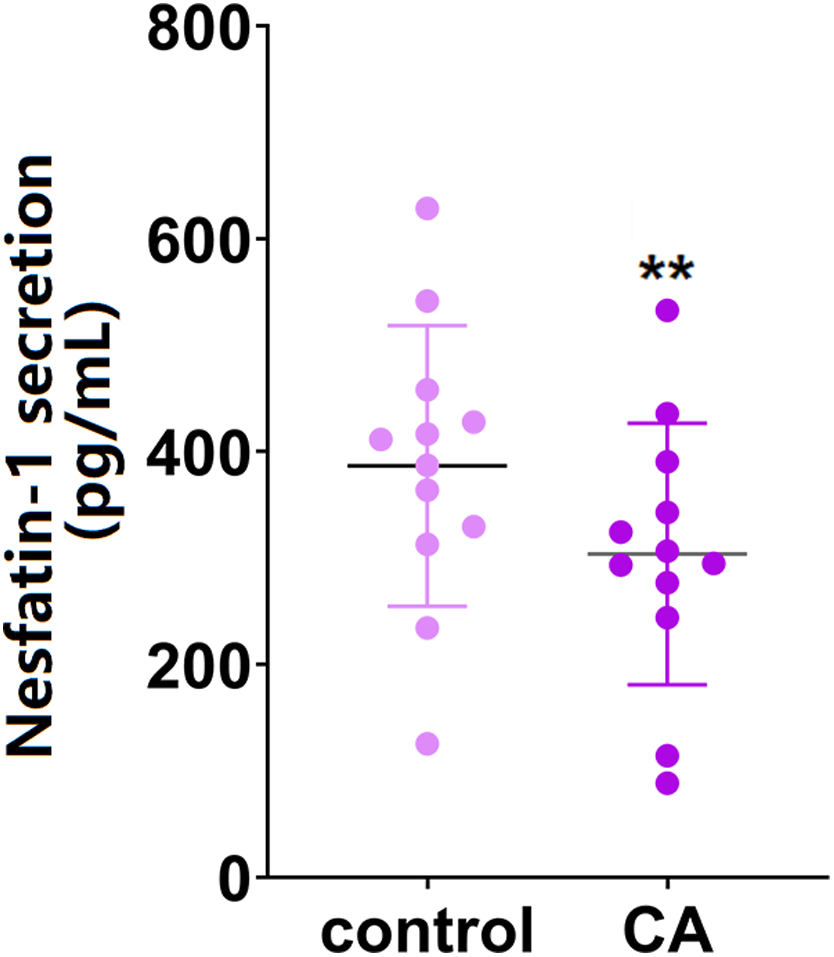
The levels of serum Nesfatin‐1 were reduced in cerebral aneurysm (CA) rats. The levels of serum Nesfatin‐1 in the control and CA rats were measured using ELISA (**, *p* < 0.01 vs. vehicle group).

To test the potential role of Nes‐1 in CA, the circulated level of Nes‐1 in CA rats was determined. Our results show that the average serum Nes‐1 level was sharply reduced from 385.6 to 303.7 pg/mL in CA rats (Figure 1), implying that lower Nes‐1 level might be associated with CA development.

### 3.2 Nes‐1 reduced the systolic blood pressure in CA rats

**FIGURE 2:**
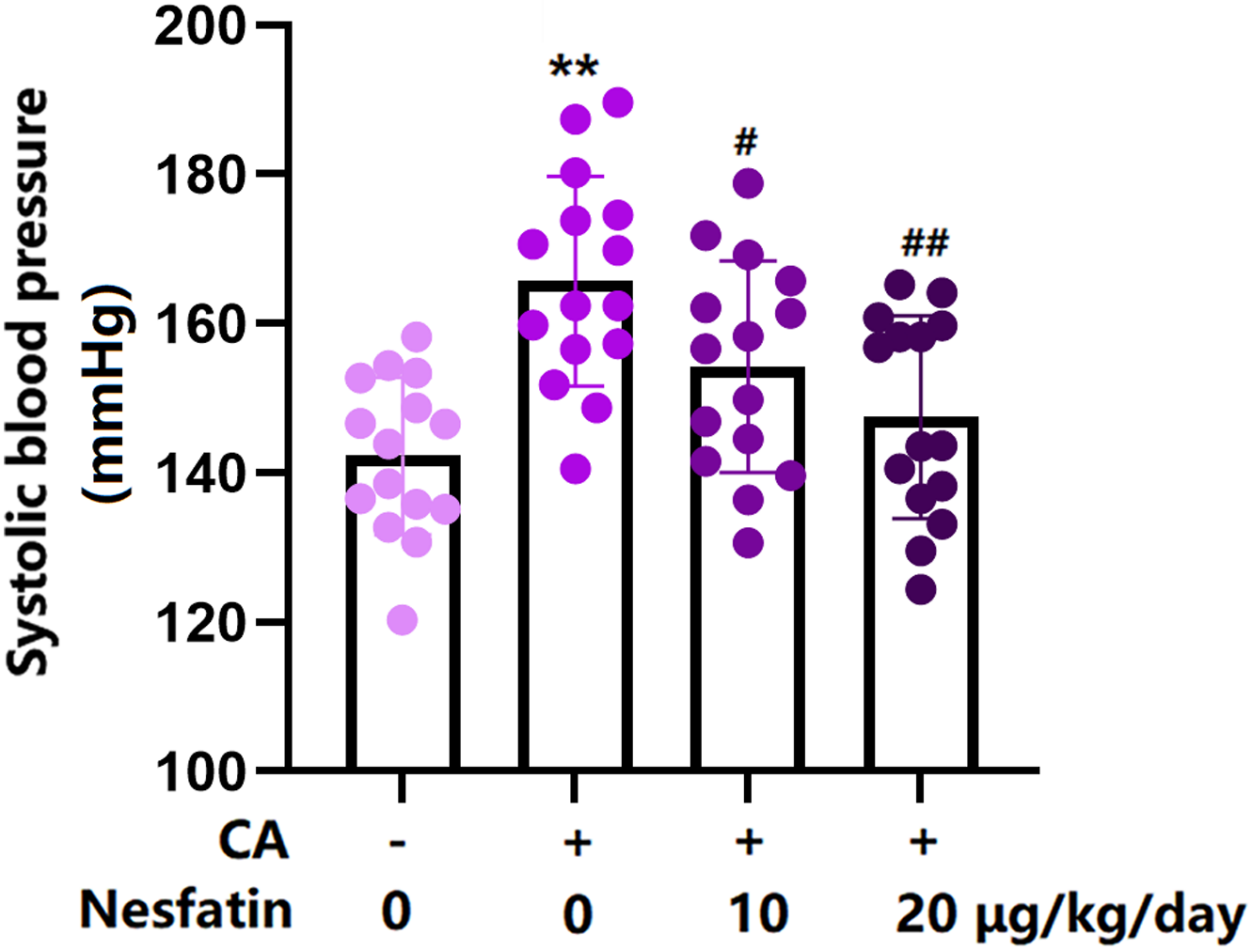
Effects of Nesfatin‐1 on systolic blood pressure in control, CA‐induced, and experimental group at 16 weeks after the surgery. Systolic blood pressure was measured (**, *p* < 0.01 vs. vehicle group, ^#^, ^##^, *p* < 0.05, 0.01 vs. CA group).

Enhanced systolic blood pressure is a critical characteristic of CA.^19^ Nes‐1 (10 and 20 μg/kg) was administered intraperitoneally to rats 1 week before the induction of CA and continued until the end of the experiment. The systolic blood pressure in the control, CA, 10, and 20 μg/kg Nes‐1 groups was 142.3, 165.7, 154.2, and 147.5 mm Hg (Figure 2), respectively, implying a repressive effect of Nes‐1 against the increased systolic blood pressure in CA rats.

### 3.3 Nes‐1 suppressed CA formation in rats

**FIGURE 3:**
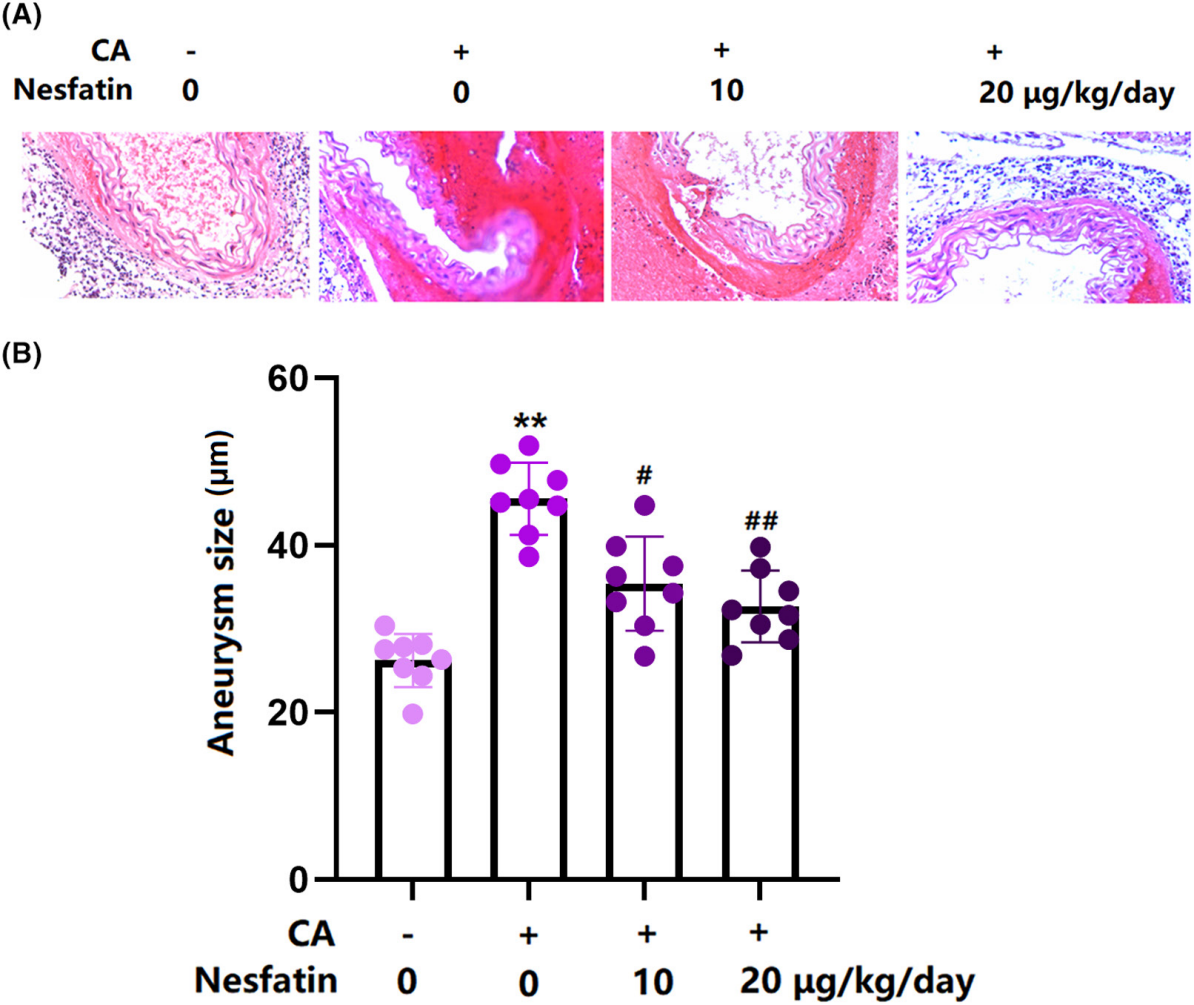
Administration of Nesfatin suppressed CA formation in rats. (A) Verhoeff‐Van Gieson staining of the vascular wall. (B) Measurement of aneurysm size (**, *p* < 0.01 vs. vehicle group, ^#^, ^##^, *p* < 0.05, 0.01 vs. CA group).

Representative images of Verhoeff‐Van Gieson staining of the vascular wall are illustrated in Figure 3A. The quantitative results of the four groups are illustrated in Figure 3B. Before the operation, the mean aneurysm size in control animals was 26.2 μm. After the CA operation, it was increased to 45.2 μm, which was notably reduced to 35.4 and 32.7 μm by the treatment with 10 and 20 μg/kg Nes‐1, respectively (Figure 3B).

### 3.4 Nes‐1 repressed the inflammatory response in the COW region of CA rats

**FIGURE 4:**
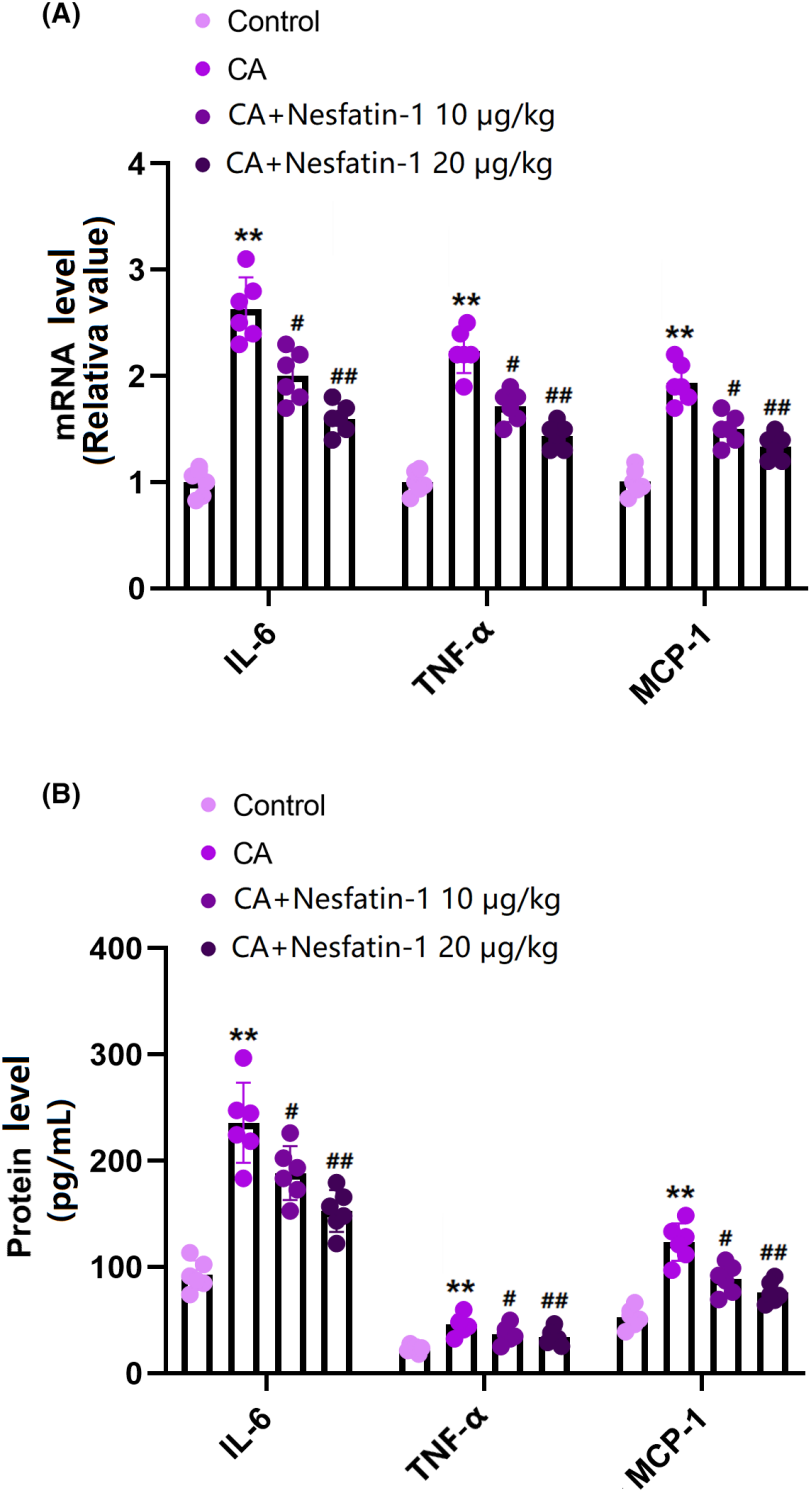
Administration of Nesfatin inhibited the expression of inflammatory cytokines IL‐6, TNF‐α, and MCP‐1 in the COW region. (A) mRNA of IL‐6, TNF‐α, and MCP‐1 as measured by real‐time PCR; (B) Protein of IL‐6, TNF‐α, and MCP‐1 as measured by ELISA (**, *p* < 0.01 vs. vehicle group, ^#^, ^##^, *p* < 0.05, 0.01 vs. CA group).

mRNA levels of IL‐6, TNF‐α, and MCP‐1 in the COW region of CA rats were sharply elevated but remarkably inhibited by 10 and 20 μg/kg Nes‐1 (Figure 4A). In addition, the average IL‐6 levels in the control, CA, 10, and 20 μg/kg Nes‐1 groups were 92.3, 235.9, 188.6, and 152.7 pg/mL, respectively. The TNF‐α content was significantly increased from 23.2 to 45.7 pg/mL in CA rats and then markedly decreased to 36.8 and 33.9 pg/mL by 10 and 20 μg/kg Nes‐1, respectively. In addition, the average MCP‐1 concentrations in the control, CA, 10, and 20 μg/kg Nes‐1 groups were 52.7, 123.5, 88.5, and 76.2 pg/mL, respectively (Figure 4B). These data indicate that the high inflammatory response observed in the COW region of CA rats was repressed by Nes‐1.

### 3.5 Nes‐1 inhibited MMP‐2 and MMP‐9 levels in the COW region of CA rats

**FIGURE 5:**
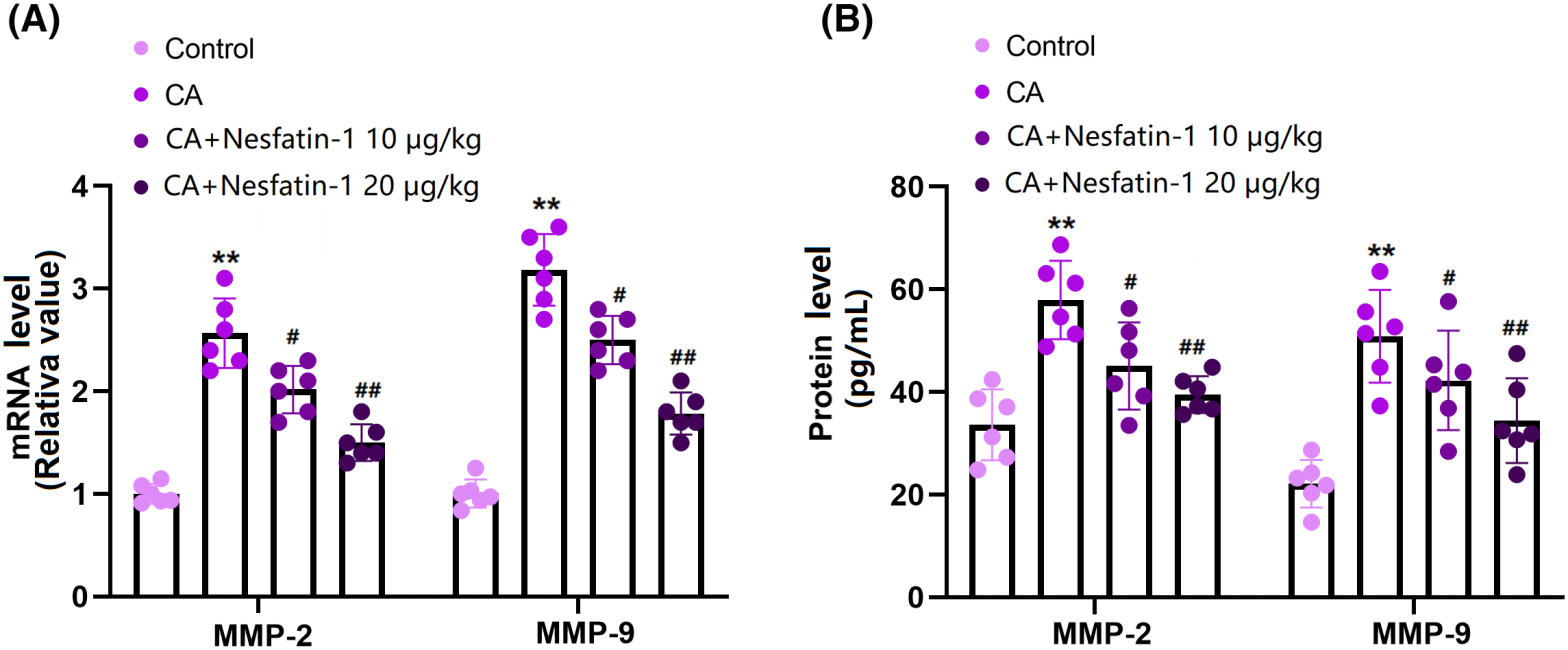
Administration of Nesfatin inhibited the expression of MMP‐2 and MMP‐9 in the COW region. (A) mRNA of MMP‐2 and MMP‐9 as measured by real‐time PCR; (B) Protein of MMP‐2 and MMP‐9 as measured by ELISA (**, *p* < 0.01 vs. vehicle group, ^#^, ^##^, *p* < 0.05, 0.01 vs. CA group).

Elevated MMP levels are reported in CA development.^20^ MMP‐2 and MMP‐9 levels in the COW region of CA rats were markedly enhanced, but signally reduced by 10 and 20 μg/kg Nes‐1 (Figure 5A). Moreover, the average MMP‐2 levels in the control, CA, 10, and 20 μg/kg Nes‐1 groups were 33.6, 56.3, 45.0, and 39.5 pg/mL, respectively. The average MMP‐9 content in CA rats was elevated from 22.1 to 50.9 pg/mL, which was notably suppressed to 40.6 and 34.5 pg/mL 10 and 20 μg/kg Nes‐1, respectively (Figure 5B).

### 3.6 Nes‐1 prevented macrophage infiltration in the COW region of CA rats

**FIGURE 6:**
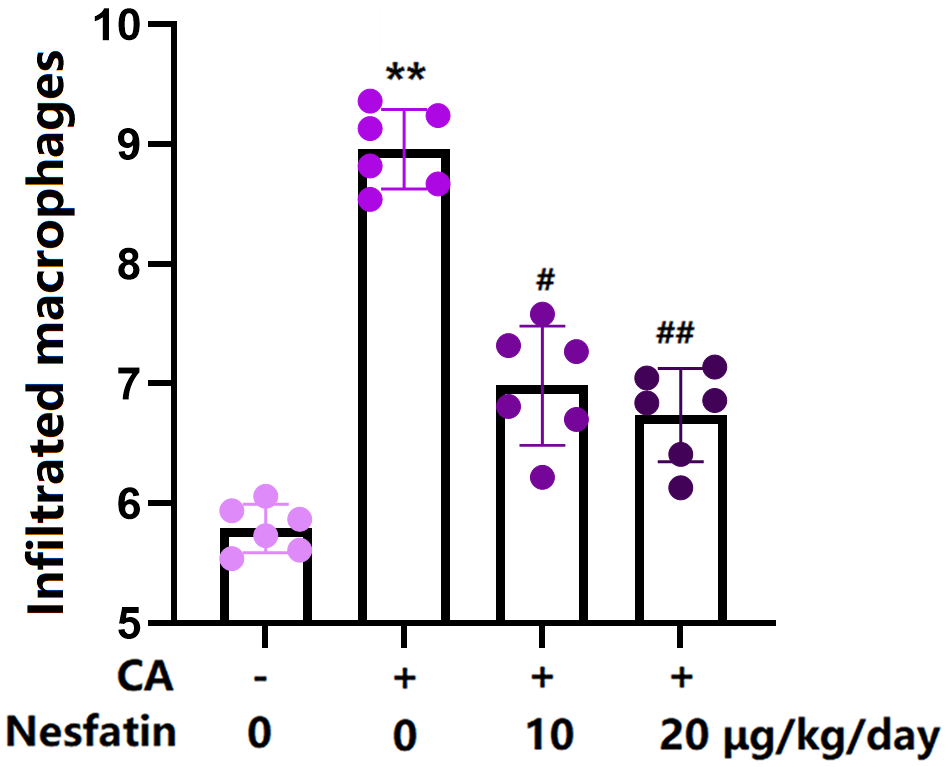
Administration of Nesfatin prevented the macrophage infiltration. The infiltrated macrophages were counted (**, *p* < 0.01 vs. vehicle group, ^#^, ^##^, *p* < 0.05, 0.01 vs. CA group).

Macrophage infiltration is reported to be responsible for the inflammatory response in CA development.^21^ Our results show that the number of infiltrating macrophages in CA rats was sharply increased, but was remarkably repressed by 10 and 20 μg/kg Nes‐1 (Figure 6).

### 3.7 Nes‐1 activated Nrf2 and NQO‐1 in the COW region of CA rats

**FIGURE 7:**
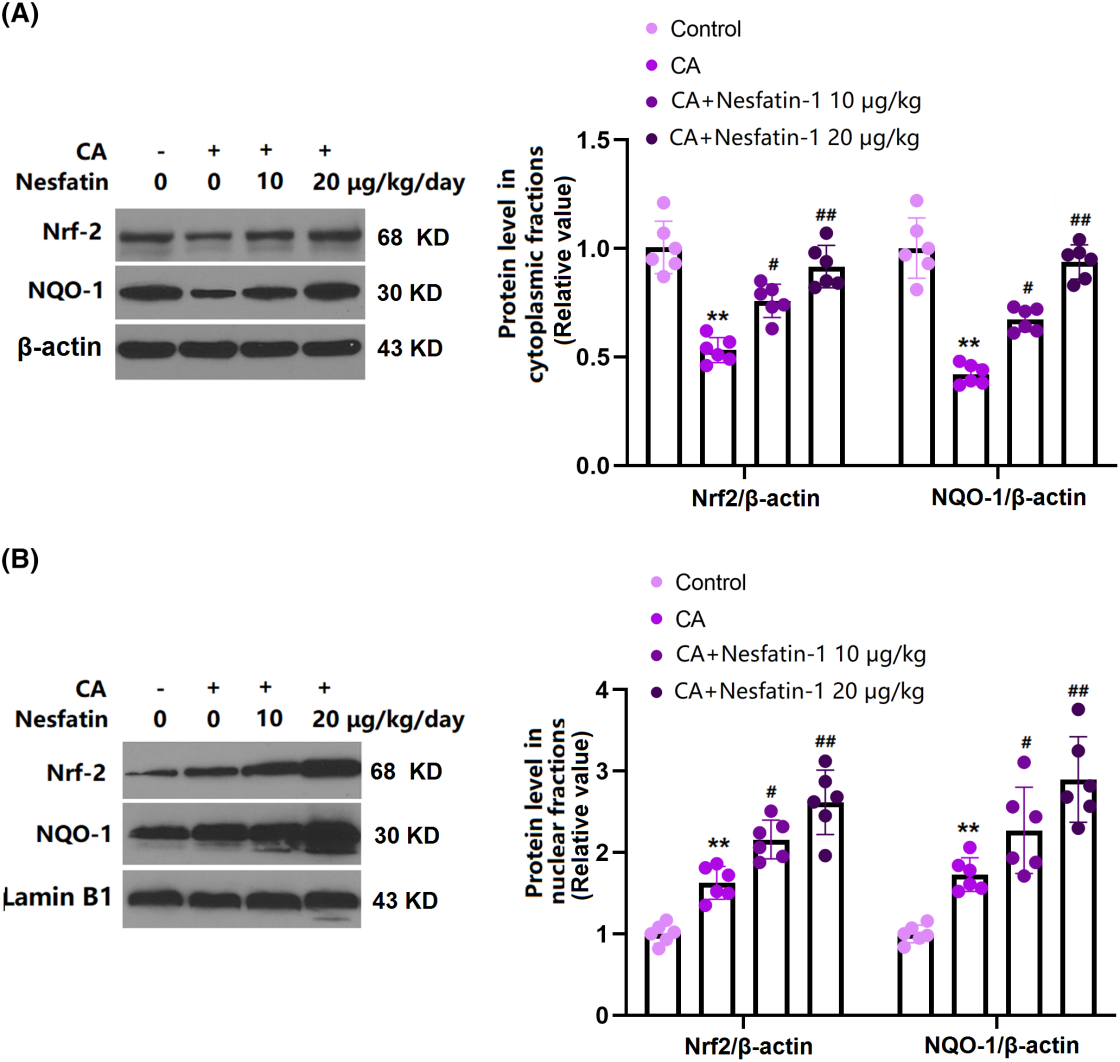
Nesfatin promoted nuclear translocation of Nrf‐2 with decreased cellular ROS levels. (A) The levels of Nrf‐2 and NQO‐1 in cytoplasmic fractions as measured by western blot analysis. (B) The levels of Nrf‐2 and NQO‐1 in nuclear fractions as measured by western blot analysis (**, *p* < 0.01 vs. vehicle group, ^#^, ^##^, *p* < 0.05, 0.01 vs. CA group).

Activation of the Nrf2/NQO‐1 axis is known to be associated with suppressed macrophage infiltration.^22^ We found that average levels of Nrf2 and NQO‐1 in cytoplasmic fractions were sharply repressed in CA rats, but were remarkably elevated by 10 and 20 μg/kg Nes‐1 (Figure 7A). Moreover, the elevated Nrf2 and NQO‐1 levels in nuclear fractions in CA rats were further enhanced by 10 and 20 μg/kg Nes‐1 (Figure 7B).

### 3.8 Nes‐1 prevented activation of the IκBα/NF‐κB pathway in the COW region of CA rats

**FIGURE 8:**
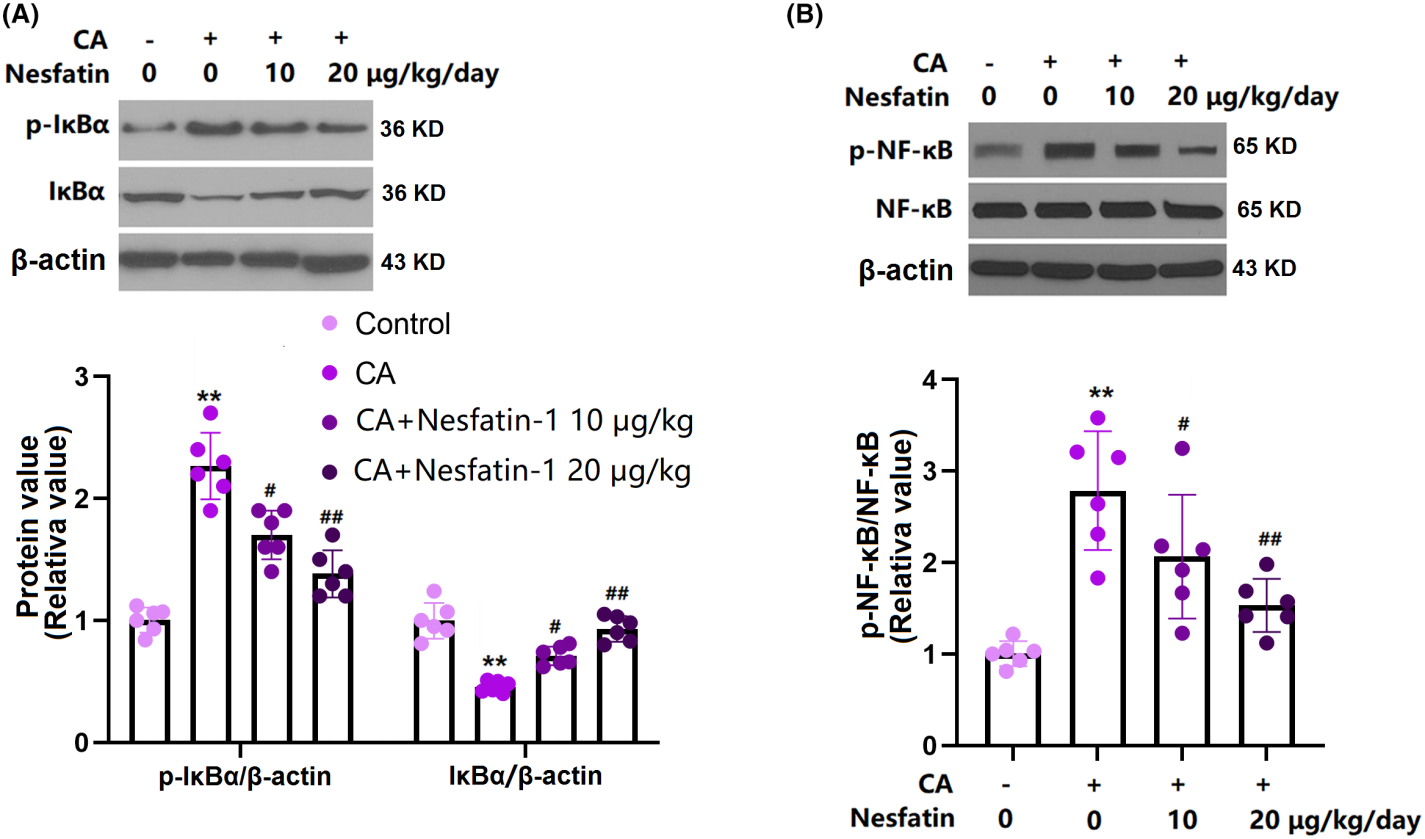
Nesfatin prevented activation of the IκBα/NF‐κB signaling pathway. (A) The levels of p‐IκBα and total IκBα. (B) The levels of p‐NF‐κB and NF‐κB (**, *p* < 0.01 vs. vehicle group, ^#^, ^##^, *p* < 0.05, 0.01 vs. CA group).

NF‐κB signaling is responsible for the activation of inflammatory response in multiple diseases.^23^ Our data show that p‐IκBα and p‐NF‐κB were sharply upregulated, while IκBα was markedly downregulated in CA rats, which were reversed by 10 and 20 μg/kg Nes‐1 (Figure 8A,B).

## 4 DISCUSSION

A very early sign of CA is endothelial dysfunction.^24^ Vessel wall damage stimulates the release of endothelial progenitor cells (EPC).^25^ Compared to the healthy control group, patients with the risk of vascular disease have reduced circulating EPC, increased endothelial cell aging, and reduced repair capacity of the vascular wall.^26^ The secretion of MCP‐1 by endothelial cells is another important event in the formation of CA. By binding to two loci on the MCP‐1 gene, NF‐κB upregulates the MCP‐1 level in endothelial cells, which contributes to the infiltration of macrophages and monocytes in the vascular wall. The infiltrating neutrophils and macrophages escalate the secretion of MCP‐1, which generates a self‐amplification loop, leading to the degradation of VSMC and ECM and promoting the development of CA.^27^ The expression of MMPs and the incidence of aneurysm are significantly decreased in MCP‐1‐knockout mice.^28^ High levels of hepatic cell growth factor (HGF) are found in CA samples, and HGF reduces levels of vascular cell adhesion molecule‐1 (VCAM‐1) and E‐selectin in endothelial cells, resulting in an effect preventing vascular inflammation.^29^ Herein, a CA model was constructed in rats and verified by increased systolic blood pressure, aneurysm size, and production of cytokines and MMPs, consistent with phenotypes of the animal models established by Wei^30^ and Aoki.^31^ Following Nes‐1 administration, declined systolic blood pressure, reduced aneurysm size, and repressed release of cytokines and MMPs were observed, implying a promising anti‐CA property of Nes‐1.

Frosen et al.^32^ conducted a histopathological analysis of human CA tissues and found that the number of macrophages infiltrating the ruptured CA tissues was higher than that of non‐ruptured CA tissues, suggesting that the increase in macrophage infiltration of the tissue occurred before the CA rupture. It is known that bone marrow‐derived monocytes are the main source of macrophages in the vascular wall of CA. The computational analysis results of human CA tissue hydrodynamics indicate that during the initial formation stage of CA, changes in intravascular hemodynamics result in increased intravascular pressure and vascular wall shear stress, damage to vascular endothelial cells, resulting in functional impairment, and release of TNF‐α, IL‐1β, MCP‐1, and VCAM‐1. Bone marrow‐derived monocytes are sensitive to the stimulation of these cytokines to migrate to the site of vascular injury, infiltrate the vascular endothelium, and differentiate into macrophages.^33^, ^34^ Macrophages directly secrete different cytokines to participate in inflammatory response by regulating other immune cells such as T cells and mast cells.^35^ In addition, macrophages secrete different levels of MMPs and tissue inhibitors of metalloproteinases (TIMPs) through different stages, resulting in an imbalance of MMPs and TIMPs in CA tissues, further leading to degradation of gelatin in the blood vessels and degradation of ECM.^36^ Herein, the enhanced release of inflammatory cytokines and MMPs in the COW region of CA rats was accompanied by increased macrophage infiltration, in line with observations in CA mice reported by Jin.^37^ After Nes‐1 administration, the macrophage infiltration was inhibited, implying that the anti‐CA function of Nes‐1 was correlated with the repression of macrophage infiltration. Furthermore, activation of the Nrf2/NQO‐1 axis was induced by Nes‐1, suggesting that the influence of Nes‐1 on macrophage infiltration was associated with the activation of Nrf2/NQO‐1 signaling, which is an important regulatory mechanism for macrophage function.^22^

NF‐κB is reported to be highly activated in human CA tissues, particularly in the intima,^38^ and its mRNA level is positively correlated with the severity of CA.^39^ Similarly, Aoki et al.^40^ found that NF‐κB in both macrophages and endothelial cells infiltrating mouse CA tissues was upregulated. Furthermore, in IκB‐overexpressed CA mice, declined expression of downstream factors of the NF‐κB signal is observed, accompanied by reduced macrophage infiltration and declined rate of CA formation, indicating that the activation of NF‐κB signaling leads to the increased expression of a large number of downstream inflammatory factors to promote inflammatory response and CA formation. As one of the key factors of macrophage polarization, an activated NF‐κB pathway promotes the M1 polarization of macrophages to induce severe inflammatory response.^41^, ^42^ Herein, in line with data reported by Ma,^43^ the activation of IκBα/NF‐κB signaling was observed in CA rats, which was sharply repressed by Nes‐1, implying that Nes‐1 inhibited the macrophage infiltration by repressing NF‐κB signaling. In the future, the regulatory function of Nes‐1 on Nrf2 and NF‐κB signaling will be further identified in in vitro macrophages.

This study has several limitations that should be acknowledged. Firstly, the administration of Nes‐1 precedes the surgical induction of cerebral aneurysms (CA) by one week, leading us to conclude that Nes‐1 possesses a potential preventive effect rather than a therapeutic effect. While ongoing research is assessing Nes‐1's therapeutic efficacy in the same CA model, further investigations are required to uncover its molecular mechanisms across different cell types, including macrophages, neutrophils, endothelial cells, and other immune cells. Secondly, despite reports of Nes‐1's effects in other disease models,^15^, ^16^, ^17^, ^18^ its precise regulatory spectrum in the context of CA remains to be fully elucidated. Additionally, given the pathological differences between chronic development of CA in humans and acute experimental induction of rodent models,^44^ clinical experiments are necessary to validate the role of Nes‐1 in human subjects.

In conclusion, this study revealed that Nes‐1 suppressed CA formation by mediating the Nrf2/NF‐κB signaling pathway in the surgically induced rodent model. Our findings imply that the adipokine Nes‐1 possesses a preventive effect on the development of cerebral aneurysms.

## CONFLICT OF INTEREST STATEMENT

There is no conflict of interest.

## Supporting information


Appendix S1.

## Data Availability

The data that support the findings of this study are available from the corresponding author upon reasonable request.
