# Supplementary material for: Nesfatin‐1 inhibits cerebral aneurysms by activating Nrf2 and inhibiting NF‐κB signaling
Source: CNS Neurosci Ther. 2024 Aug 4;30(8):e14864. doi: 10.1111/cns.14864 (PMC11298201; doi:10.1111/cns.14864)

Full unedited blot for Figure 7A

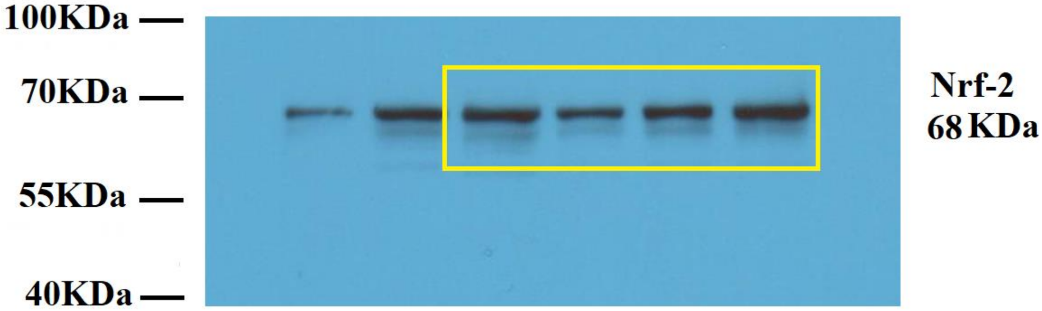

Full unedited blot for Figure 7A

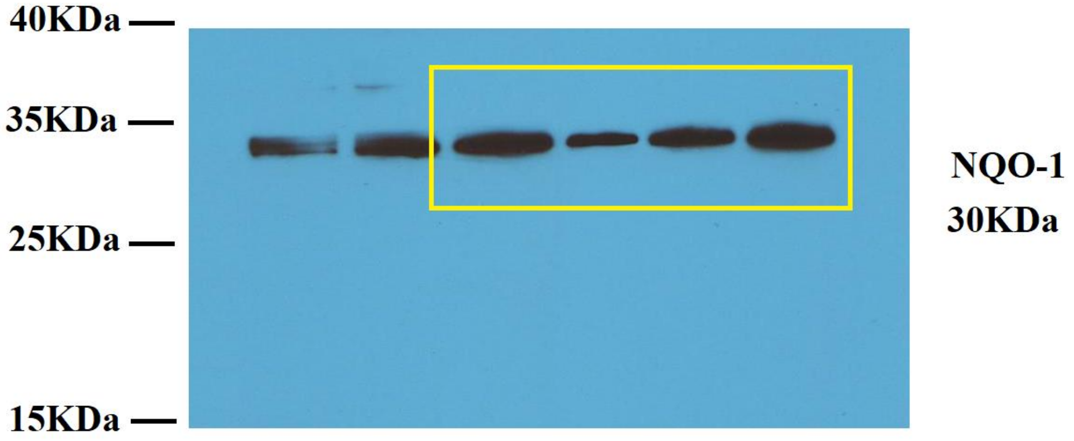

Full unedited blot for Figure 7A

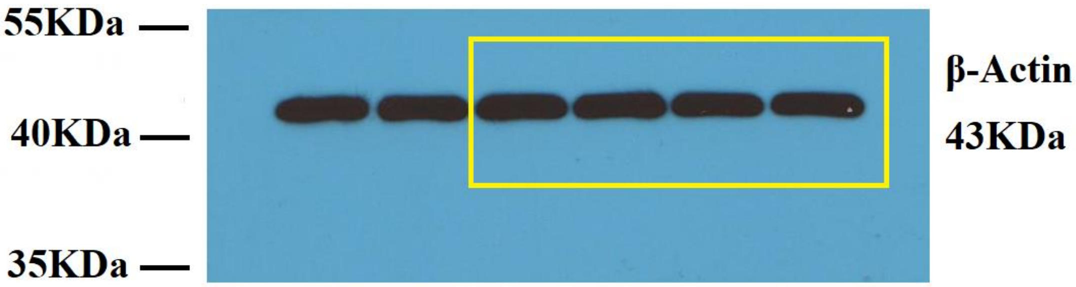

Full unedited blot for Figure 7B

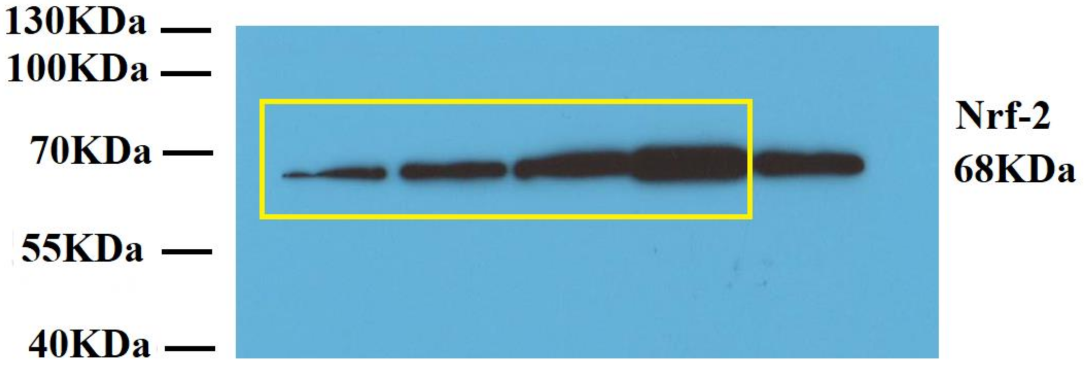

Full unedited blot for Figure 7B

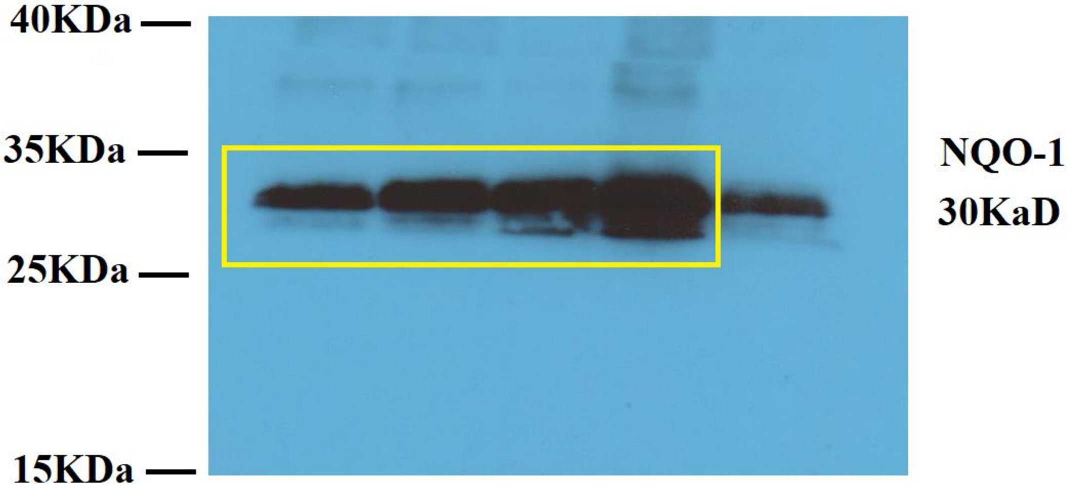

Full unedited blot for Figure 7B

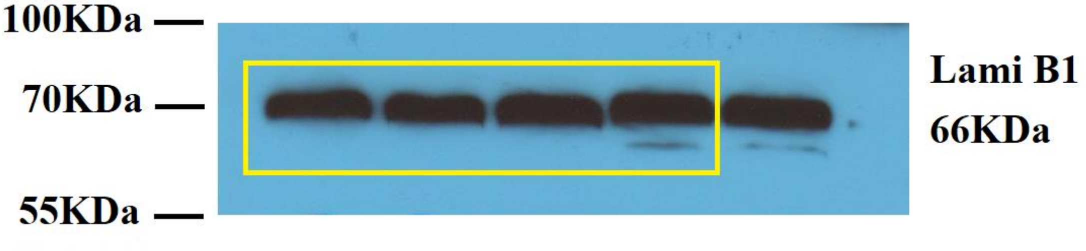

Full unedited blot for Figure 8A

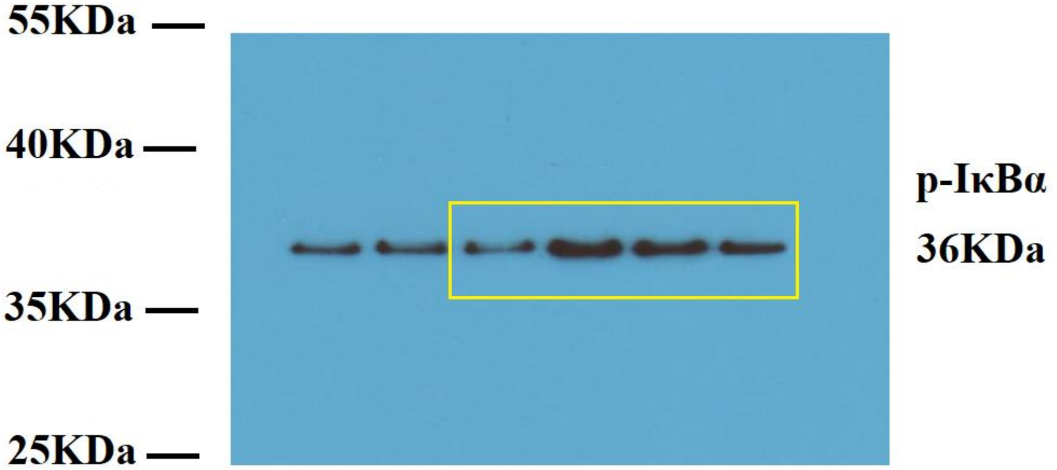

Full unedited blot for Figure 8A

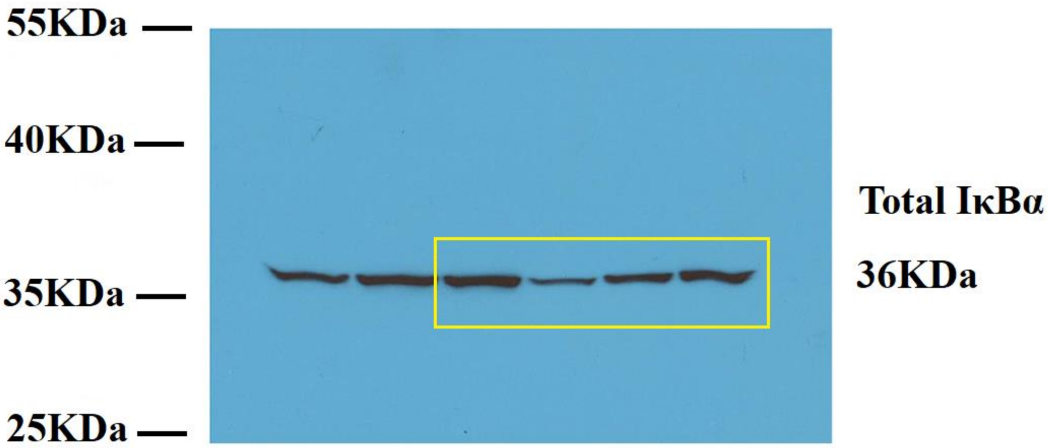

Full unedited blot for Figure 8A

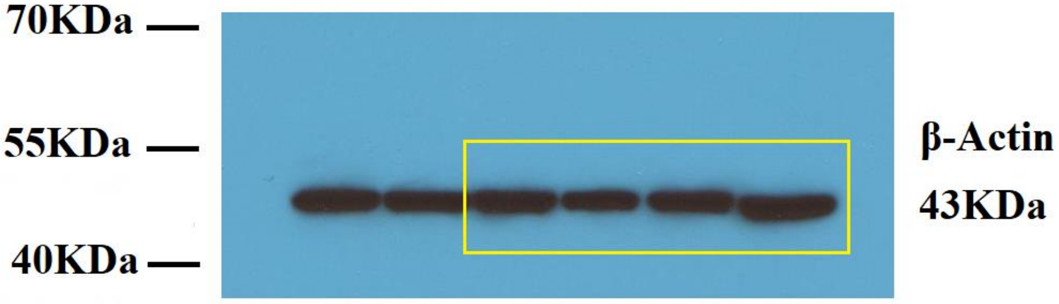

Full unedited blot for Figure 8B

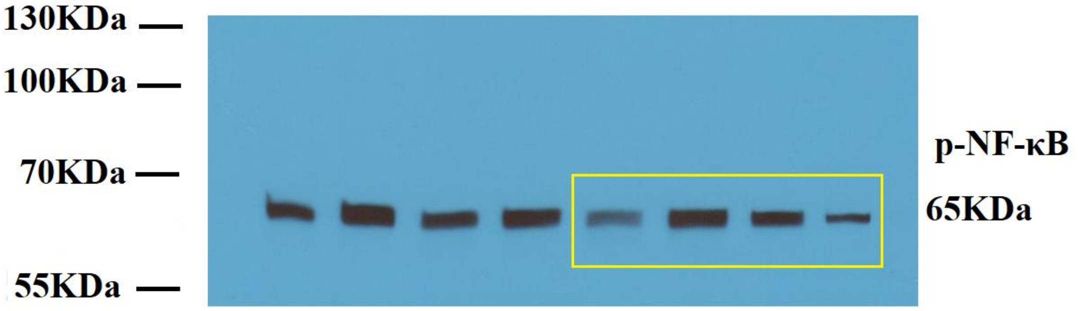

Full unedited blot for Figure 8B

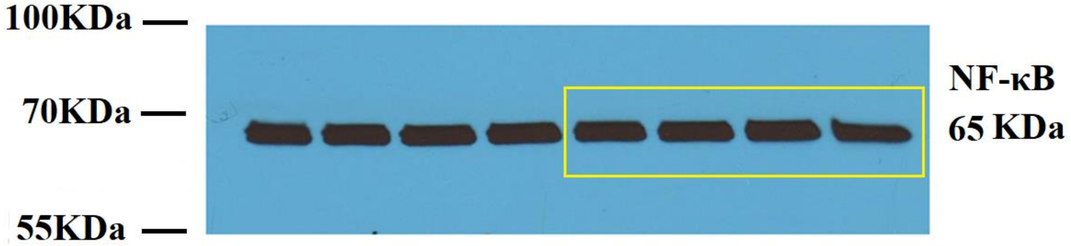

Full unedited blot for Figure 8B

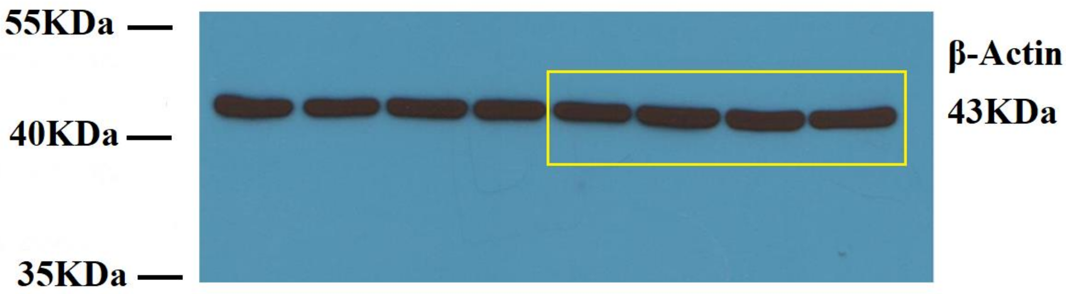

Supplement: Supplementary file 1 — Appendix S1. [file CNS-30-e14864-s001.pdf]
